# Cytokines, chemokines and leukotrienes profile and signature analysis in HTLV-1 infection as an evidence of disease progression

**DOI:** 10.1186/1742-4690-11-S1-O37

**Published:** 2014-01-07

**Authors:** Ana LB Starling, Denise U Gonçalves, Vanessa Peruhype-Magalhães, Jordana Coelho-dos-Reis, José R Lambertucci, Ludimila Labanca, Silvio R Souza Pereira, Marina L Martins, João G Ribas, Andréa Teixeira-Carvalho, Bruno C Trindade, Lucia H Faccioli, Anna BF Carneiro-Proietti, Olindo A Martins-Filho

**Affiliations:** 1 Curso de Pós-Graduação em Infectologia e Medicina Tropical,Faculdade de Medicina da Universidade Federal de Minas Gerais - Belo Horizonte, Minas Gerais, Brazil; 2 Laboratório de Biomarcadores de Diagnóstico e Monitoração, Centro de Pesquisa René Rachou, FIOCRUZ-Minas, Belo Horizonte, Minas Gerais, Brazil; 3 Fundação Centro de Hematologia e Hemoterapia de Minas Gerais, HEMOMINAS, Minas Gerais, Brazil; 4 Hospital Sarah Kubitschek, Belo Horizonte, Minas Gerais, Brazil; 5 Faculdade de Farmácia da USP, Ribeirão Preto, São Paulo, Brazil

## Background

A cross-sectional study evaluated the cytokines, chemokines and leukotrienes profiles as possible biomarkers of progression to HTLV-1 associated myelopathy (HAM).

## Methods

Serum samples from 21 healthy blood donors (HBD), 27 asymptomatic carriers (ASC), 32 possible HAM (pHAM) and 28 HAM individuals were tested for cytokines (IL-6, IFN-γ, TNF-α, IL-2, IL-4 and IL-10), chemokines ( RANTES, MCP1, IL-8, MIG and IP-10) and leukotrienes (CysLTs and LTB-4). For each molecule tested, the HTLV-1 individuals were classified as low or high-producers taking the global median index of the HBD group as a cut off.

## Results

When comparing AS and pHAM individuals, AS were high-producers of IP-10 and low-producers of RANTES; pHAM were high-producers of IL-2 and low of IL-8. Besides, AS individuals presented a strong positive correlation between the regulatory cytokines IL-10 with IL-4 and between both with the pro-inflammatory cytokines IL-2 and IL-6; a negative correlation was found between RANTES and IL-2. HAM were high-producers of IL-6, IFN-γ, IP-10, LTB4, IL-4, MIG, IL-10, IL-2, presented a positive correlation of TNF-α and IFN-γ with IL-6, but this group had a positive correlation of CysLT with IL-10, IL-4 and TNF-α, contrasting with other groups.

## Discussion

HAM displayed a unique signature of inflammation, which was strengthened by CysLT and not counterbalanced by IL-4 and IL-10. This signature was observed in pHAM to a lower extent, becoming more evident in HAM. This profile may indicate disease progression and may serve as prognostic markers in future studies.

